# Supplementary material for: The genome sequence of an alderfly, Sialis fuliginosa Pictet, 1836
Source: Wellcome Open Res. 2024 Jun 19;9:321. [Version 1] doi: 10.12688/wellcomeopenres.22460.1 (PMC11549539; doi:10.12688/wellcomeopenres.22460.1)
Supplement: Peer review history

# Reviewer report: Reviewer response for version 1

**DOI:** 10.21956/wellcomeopenres.24747.r106605

**Published:** 2024-11-08

**Authors:** Xinglong Huang

**Affiliations:** 1 Jishou University, ishou, Hunan, China

This paper reports the genome of the alderfly, *Sialis lutaria*. This is novel and important information regarding a Megaloptera species and may prove useful bioinformatic and molecular data for future phylogenetic and molecular studies on this species. Methods are clear and thoroughly explained. The discussions and results are clear and insightful. I recommend indexing.

Are sufficient details of methods and materials provided to allow replication by others?

Yes

Is the rationale for creating the dataset(s) clearly described?

Yes

Are the datasets clearly presented in a useable and accessible format?

Yes

Are the protocols appropriate and is the work technically sound?

Yes

Reviewer Expertise:

Aquatic insect

I confirm that I have read this submission and believe that I have an appropriate level of expertise to confirm that it is of an acceptable scientific standard.

# Reviewer report: Reviewer response for version 1

**DOI:** 10.21956/wellcomeopenres.24747.r105218

**Published:** 2024-10-28

**Authors:** Marta Coronado-Zamora

**Affiliations:** 1 Botanical Institute of Barcelona (Ringgold ID: 54411), Barcelona, Catalonia, Spain

This data note describes a high quality genome assembly of an alderfly, *Sialis fuliginosa*, **using appropriate sequencing and assembly methods. The genome data are available at ENA, and data is accessible  (as checked for some of the chromosomes/datasets). 

**Comments:**

- RNA, genome and Hi-C sequencing is generated in each case in a different tissue (abdomen, thorax, head). Could this have an impact in the generation of the genome assembly? Why the RNA-seq was produced on a single tissue instead on whole-bodies if is it going to use later on for gene annotation?  
- Mention the sex determination of this specifies could be useful
- The specimens collected for Hi-C and RNA-seq were also female individuals?
- "bwa-mem 2" is missing from Table 3

Overall, this new genome resource by the Darwin Tree of Life Consortium is excellent.

Are sufficient details of methods and materials provided to allow replication by others?

Yes

Is the rationale for creating the dataset(s) clearly described?

Yes

Are the datasets clearly presented in a useable and accessible format?

Yes

Are the protocols appropriate and is the work technically sound?

Yes

Reviewer Expertise:

population genomics, transposable elements, bioinformatics, NGS analysis

I confirm that I have read this submission and believe that I have an appropriate level of expertise to confirm that it is of an acceptable scientific standard.
